# First report of occult hepatitis B infection among ART naïve HIV seropositive individuals in Maputo, Mozambique

**DOI:** 10.1371/journal.pone.0190775

**Published:** 2018-01-10

**Authors:** Awa Abdul Carimo, Eduardo Samo Gudo, Cremildo Maueia, Nédio Mabunda, Lúcia Chambal, Adolfo Vubil, Ana Flora, Francisco Antunes, Nilesh Bhatt

**Affiliations:** 1 Internal Medicine Department, Maputo Central Hospital, Maputo, Mozambique; 2 Instituto Nacional de Saúde, Ministry of Health, Maputo, Mozambique; 3 Instituto de Saúde Ambiental (ISAMB), Faculty of Medicine University of Lisbon, Lisbon, Portugal; CEA, FRANCE

## Abstract

**Background:**

The prevalence of hepatitis B virus (HBV) infection and human immunodeficiency virus (HIV) infection in Mozambique is one of the highest in the world, though in spite of this the prevalence of occult hepatitis B infection (OBI) is unknown.

**Objectives:**

This study was conducted with the aim to investigate the prevalence of OBI and frequency of isolated hepatitis B core antibody (anti-HBc alone) among antiretroviral (ART) naïve HIV-positive patients in Mozambique.

**Methods:**

A cross-sectional study was conducted in two health facilities within Maputo city. All ART-naive HIV seropositive patients attending outpatient clinics between June and October 2012 were consecutively enrolled. Blood samples were drawn from each participant and used for serological measurement of HBV surface antigen (HBsAg), antibodies against HBV surface antigen (anti-HBs) and antibodies against core antigen (anti-HBc) using ELISA. Quantification of HBV DNA was performed by real time PCR. A questionnaire was used to obtain demographics and clinical data.

**Results:**

Of the 518 ART-naive HIV-positive subjects enrolled in the study, 90.9% (471/518) were HBsAg negative. Among HBsAg negative, 45.2% (213/471) had isolated anti-HBc antibodies, and the frequency of OBI among patients with anti-HBc alone was 8.3% (17/206). OBI was not correlated either with CD4^+^ T cells count or transaminases levels. A total of 11.8% of patients with OBI presented elevated HBV DNA level. Frequency of individuals with APRI score > 2 and FIB-4 score > 3.25 was higher in patients with OBI as compared not exposed, immune and anti-HBc alone patients.

**Conclusion:**

Our data demonstrate for the first time that OBI is prevalent among HIV patients in Mozambique, and will be missed using the commonly available serological assays that measures HBsAg.

## Introduction

Occult hepatitis B virus (HBV) infection (OBI) is defined as persistence of HBV DNA in the liver (with or without HBV DNA in serum) in the absence of detectable HBV surface antigen (HBsAg) [1]. In these patients, serum HBV DNA level is usually very low, mostly below 10^4^ IU/mL. The clinical implications of OBI are not fully understood, but include: i) risk of HBV transmission through blood transfusion, hemodialysis, organ and bone marrow transplantation, and perinatal route due to the hidden status of HBV infection; ii) hepatitis B virus reactivation (HBVr) after immunosuppression; iii) risk of progression to chronic liver disease (CLD); iiii) risk of development of hepatocellular carcinoma (HCC) [1].

The underlying mechanism leading to the absence of HBsAg in presence of HBV is not completely understood. However, several mechanisms such as: 1./ presence of a strong humoral and cellular immune response against HBV envelope proteins, leading to a low level of HBV replication and low level of HBsAg expression; 2./ escape mutations in the S gene leading to lack of detection of the virus using HBsAg assays; 3./ infection with a mutant virus with defective replication; 4./ formation of HBsAg/anti-HBs immunocomplexes which prevent detection of HBsAg. and 5./ integration of HBV genome into the host chromosomes, infecting peripheral mononuclear cells (monocytes and lymphocytes) that works as reservoirs. Finally, the coinfections with other viruses such as hepatitis C virus (HCV) and human immunodeficiency virus (HIV), or administration of drugs, including antiretrovirals, can also interfere with the HBV replication and its pathogenic features[1–4]. OBI reactivation sometimes occurs during an immunosuppression status (i.e., hematologic malignancies, chemo- or immunotherapies, hematopoietic stem cell transplantation, or HIV infection).

Several studies have shown that the frequency of OBI is higher in HIV infected as compared to HIV negative individuals, ranging between 0% and 89.5% [5–7]. The clinical impact of OBI in HIV-infected subjects is still controversial [8], but several authors suggests that the risk of hepatic flares, hepatic failure and liver cirrhosis or hepatic carcinoma is higher among these patients [1, 7–10]. Current World Health Organization (WHO) guidelines recommends that all HIV infected patients should be routinely screened for HBV and antiretroviral treatment (ART) should be initiated immediately to those co-infected with HBV, irrespective of their CD4^+^ T cells count [11]. Detection of serum HBV DNA using highly specific and sensitive nucleic acid amplification test (NAAT) is the recommended method for detection of HBV infection in clinical practice [12]. However, HBV NAAT is not available in limited resource settings, including sub-Saharan Africa, where detection of HBsAg is the most commonly used and available method for HBV screening. In this context, most HIV infected patients with OBI will remain undetected, which will severely compromise their care [13]. The presence of anti-HBc alone (defined as presence of anti-HBc antibodies in absence of other serological marker of HBV) is considered a less than ideal predictor of OBI [1] and in places where availability of molecular tests is very limited, anti-HBc has been used to prioritize samples that could be further tested by PCR [1, 13]. However, accuracy of anti-HBc alone to identify OBI had been a matter of debate as false anti-HBc positivity and negativity in the detection of OBI has been previously reported [14] and described in the Taormina statement as well [1].

In Mozambique, which has the eighth highest HIV prevalence in the world [15], the most recent HIV survey showed that the epidemics is getting even worse [16]. Few studies have been conducted to determine the prevalence of HBV in Mozambique, and they have been conducted in specific groups such as refugees [17], blood donors [18–20], HIV infected individuals [21, 22] or high risk groups [23] and showed that the rate varied between 7% and 13%. OBI has received new attention as a serious public health problem. However, no study has yet been conducted in Mozambique to investigate the epidemiology and clinical implication of OBI. Lack of data on OBI represents a major challenge to the implementation of interventions to prevent complications of co-infection. This study was conducted with the aim to investigate the frequency and clinical and laboratory characteristics of OBI in ART naïve HIV infected patients in Maputo city. This study uses the same cohort of patients of our recently published manuscript on the frequency and characteristics of HBV/HIV co-infection in Maputo [22].

## Material and methods

### Setting and population

A cross-sectional study was carried out between June and October 2012 in two public health centers from Maputo city, Mozambique. During the study period, all ART-naive HIV positive patients attending their routine follow up visits were consecutively invited to participate in this study. All participants were informed of the study and consented prior to enrollment. Inclusion criteria included age more than 18 years old and no prior history of ART. All pregnant women were excluded. All participants were assigned a unique study identification code with subsequent data collected and analyzed. The study was approved by the National Bioethics Committee in Mozambique (Ref#: 82/CNBS/12). All investigation had been conducted according to the principles expressed in Declaration of Helsinki. Oral and written informed consents were obtained from all patients or their legal guardians.

### Sample collection and testing

**Fig 1:**
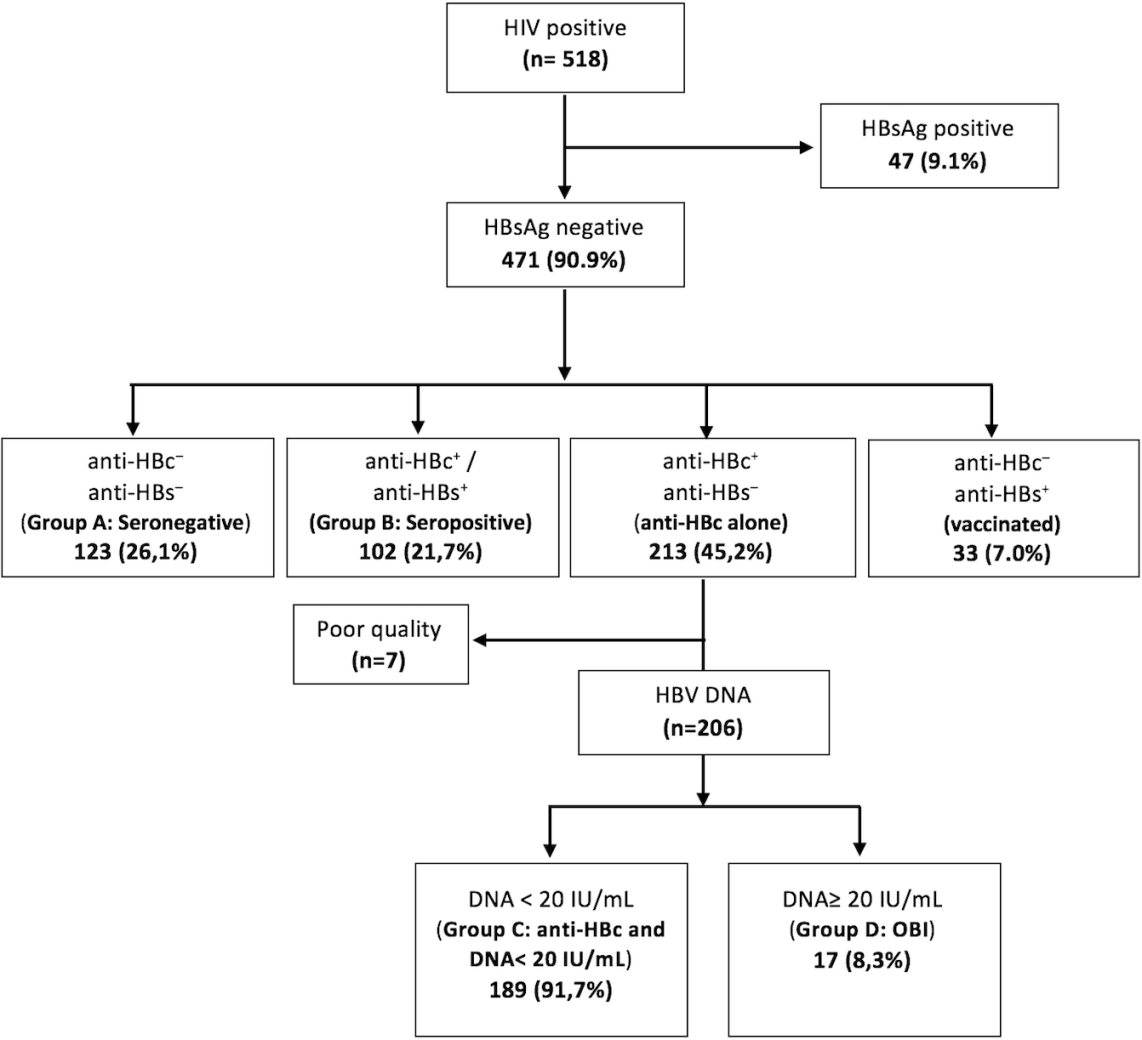
Flowchart of recruitment and testing of study participants. anti-HBc- − antibody against HBV core antigen. anti-HBs- − antibody against HBV surface antigen. DNA–desoxirribonucleic acid. HBsAg- HBV surface antigen. HBV–hepatitis B virus. HIV–human immunodeficiency virus. OBI- occult hepatitis B virus infection.

From each participant, a total of 10 ml of whole blood was collected into 5 ml of Vacuum tube with K3EDTA and 5 mL of serum separation Vacuum tube (both from BD Vacutainer, USA). Full blood counting was performed using an automated hematology analyzer (Sysmex KX21N: Sysmex Corporation, Japan). Serum transaminases were measured using a clinical chemistry analyzer (ABX pentra 400: Horiba ABX SAS, France). CD4+ T cells count was measured by flow cytometer using a lyse-no wash protocol with MultiTest reagents and FACSCalibur flow cytometer (all from Becton Dickinson, CA, USA). Testing for Hepatitis B was performed following the algorithm depicted in Fig 1. All patients were screened for HBsAg using a immunochromatographic rapid test with a sensitivity and specificity of 95.16% and 99.95% (Determine, Allere, Japan) and those with negative HbsAg result were subsequently tested for anti-HBs and anti-HBc antibodies using commercially available Enzyme Linked Immunoabsorbent Assay (ELISA) (Diasorin S.p.A., Sallugia, Italy). Due to limitation of resources, HBV DNA was prioritized and measured in all patients who were HBsAg negative, anti-HBs negative and anti-HBc positive (anti-HBc alone), using the AmpliPrep/cobas TaqMan HBV test, v2.0 (Roche Diagnostics, Germany) with a limit of detection of 20 IU/mL. In this context, OBI was defined as presence of HBV DNA in patients with serological profile of anti-HBc alone (HBsAg negative, anti-HBs negative and anti-HBc positive).

### Questionnaire

A questionnaire was used to collect socio-demographic characteristics, and clinical history from each participant (See S1 File).

### Statistical analysis

Data was double entered in a database developed using Microsoft Access 2007. Analysis was performed using the statistics package STATA 12.0 (StataCorp, College Station, TX, USA). For our analysis, HBsAg negative patients were stratified into four groups according to a combination of HBV serological and molecular markers: i) Group A–anti-HBs negative and anti-HBc negative (not exposed); ii) Group B–anti-HBs positive and anti-HBc positive (exposed and immune); iii) Group C–anti-HBs negative, anti-HBc positive and HBV DNA < 20 UI/mL (anti-HBc alone with HBV DNA < 20 UI/mL), and iv) Group D–anti-HBs negative, anti-HBc positive and viral load >20UI/mL (occult hepatitis B).

We calculated the scores of FIB-4 (Fibrosis 4 index) and APRI (AST-Platelet Ratio Index) to predict significant fibrosis and cirrhosis. These non-invasive serum biomarkers were calculated using the formula described previously [24]. As per WHO guidelines, an APRI score > 2.0 indicates significant liver fibrosis and cirrhosis, while a FIB-4 score > 3.25 indicates significant cirrhosis [11].

For univariate analysis, the Mann-Whitney U test was used to compare numerical variables and Pearson’s chi-square test was used to compare categorical variables.

## Results

The study population consisted of 518 HIV-infected patients with a median age of 33 years old, 67.0% were female, and the median CD4^+^ T cells count was 362 cells/mm^3^. Among them, 47 (9.1%) were HBsAg positive, and 471 (90.9%) were HBsAg negative. Among the HBsAg negative, 26.1% (123/471) were anti-HBs and anti-HBc negative (Group A–not exposed), 21.7% (102/471) were anti-HBs and anti-HBc positive (Group B–exposed and immune), 45.2% (213/471) were anti-HBs negative and anti-HBc positive (anti-HBc alone) and 7.0% (33/471) were anti-HBs positive and anti-HBc negative (vaccinated). The last group was excluded from our analysis. (See Fig 1).

Demographic, clinical and laboratorial characteristic of the study groups are presented in the Table 1. We noted that there was a female dominance in all study groups, and the median age was similar among all groups. Clinical signs of liver disease (jaundice, ascites, splenomegaly and hepatomegaly) were not observed in any groups (See S2 File). We noted that the stratification by WHO clinical stage was similar for all groups. All groups were also similar in terms of laboratory findings, such as total leukocytes, total lymphocytes and CD4^+^ T cells counts.

**Table 1:** Demographic, clinical and laboratorial characteristic in HIV patients with HBsAg negative.

Characteristic	Group A	Group B	Group C	Group D	p-value
	Seronegative-	Seropositive-	anti-HBc^+^alone	OBI	
	anti-HBs^–^/anti-HBc^–^	anti-HBs^+^/anti-HBc^+^	anti-HBs^–^/anti-HBc^+^/(DNA<20 IU/mL)	anti-HBs^–^/anti-HBc^+^/(DNA≥20 IU/mL)	
**Total, n (%)**	**123 (26.1)**	**102 (21.7)**	**189 (40.1)**	**17 (8.3)**	
**Female (%)**	79 (64.8)	63 (62.4)	128 (68.1)	13 (76.5)	0.567
**Median age, years (IQR)**	32 (27–41)	35 (29–42)	35 (28–43)	31 (27–39)	0.428
**WHO clinical stage (%)**					
Stage I	55 (45.5)	37 (37.4)	93 (50.3)	5 (31.3)	0.535
Stage II	34 (28.1)	33 (33.3)	55 (29.7)	6 (37.5)	
Stage III	30 (24.8)	29 (29.3)	36 (19.5)	5 (31.3)	
Stage IV	2 (1.7)	0 (0.0)	1 (0.6)	0 (0.0)	
**ALT (IU/L) (IQR)**	21,8 (16.5–30.7)	21,4 (14.6–30.1)	21,7 (16.6–30.9)	24,7 (16.6–49.7)	0.493
**APRI**					
< = 2.0	121 (98.4)	99 (98.0)	187 (98.9)	14 (87.5)	0.017
>2.0	2 (1.6)	2 (2.0)	2 (1.1)	2 (12.5)	
**FIB-4**					
< = 3.25	120 (98.4)	98 (98.0)	187 (99.5)	15 (93.7)	0.309
>3.25	2 (1.6)	2 (2.0)	1 (0.5)	1 (6.3)	
**Leucocyte count (10**^**3**^ **cells/mm**^**3**^**) (IQR)**	4,7 (3.8–5.7)	4,5 (3.7–5.5)	4,8 (3.8–5.8)	4,3 (3.7–5.3)	0.581
**Lymphocyte count (10**^**3**^ **cells/mm**^**3**^**) (IQR)**	2 (2–2)	2 (1–2)	2 (1–2)	2 (1–2)	0.190
**CD4**^**+**^**T cell count (cells/mm3) (IQR)**	391 (211–538)	322 (204–477)	355 (202–517)	334 (86–543)	0.457

ALT–alanine aminotransferase; anti-HBc^−^− antibody against HBV *core* antigen; anti-HBs^−^− antibody against HBV surface antigen; CD4^+^ T–lymphocyte T with CD4^+^ phenotype; DNA–desoxirribonucleic acid; HBsAg–HBV surface antigen; HBV–hepatitis B virus; HIV–human immunodeficiency virus; IQR–interquartile range; IU/L–International Units per litre; OBI–occult hepatitis B virus infection; WHO–World Health Organization

HBV DNA quantification was performed in a total of 206 anti-HBc alone patients (seven samples were excluded due to poor quality), of which 17 were HBV DNA positive, yielding a prevalence of 8.3% (Group D–OBI). The remaining 189 (40.1%) had an undetectable serum level of HBV DNA (anti-HBc alone with HBV DNA < 20 UI/mL) (Fig 1). In patients with OBI, 64.7% (11/17), 23.5% (4/17) and 11.8% (2/17) had a viral load of 20–100 IU/mL, 101–1000 IU/mL and > 1000 IU/mL, respectively (Table 2).

**Table 2:** Serum viral load (DNA of HBV) in 17 HIV patients, with occult hepatitis B infection.

HBV viral load (UI/mL)	Number of samples	%
20–100	11	64,7
101–1.000	4	23,5
>1.000	2	11,8

DNA–desoxirribonucleic acid; HBV–hepatitis B virus; HIV–human immunodeficiency virus; IU/mL–International Units per milliliter; Compared to other groups, patients with OBI presented a higher frequency of APRI score >2 (12.5% in OBI versus 1.6% in not-exposed, 2.0% in immune and 1.1% in anti-HBc alone, p-value = 0.017) and FIB-4 score > 3.25 (6.3% in OBI versus 1.6% in not-exposed, 2.0% in immune and 0.5% in anti-HBc alone, p-value = 0.309).

## Discussion

In this study we used the same cohort of HIV infected patients of our recently published manuscript on HIV/HBV co-infection [22] to further investigate occurrence of OBI, and we found a prevalence of OBI of 8.3% in anti-HBc alone HIV infected patients, which was similar to that reported in other studies, but higher and lower frequencies had also been described elsewhere [25–31]. The differences in the prevalence of OBI in different studies might be attributed to: i) differences in the epidemiology of both viruses, ii) differences in limit detection of different PCR assays, iii) differences in the study design and iv) differences in the definition criteria of OBI in different studies [9, 31]. We acknowledge that our criteria of OBI may have led to under or over estimation of OBI, because current OBI definition criteria recommend serial testing per patient and use of PCR assays targeting two different HBV genomic places. However, due to a lack of resources, in this study only patients with anti-HBc alone patients were tested and we used a PCR assay targeting only one place of HBsAg gene which was tested in only one sample per patient. This algorithm could have missed some cases and we acknowledge this as a limitation of our study.

To our knowledge this represents the first report of OBI in Mozambique and demonstrates that OBI is prevalent among HIV/HBV co-infected Mozambicans, suggesting that presence of HBV infection among HIV infected individuals will not be identified in a significant number of co-infected patients when screened using the routinely used serological tests based on measurement of HBsAg. Of note, if we add the number of patients with OBI (n = 17) reported in this study with those already known to be HBV positive, based on measurement of HBsAg who were reported in our previous publication using this cohort of patients (n = 47) [22], we conclude that the more accurate number of HBV infected patients in this group of patients is 64, corresponding to a prevalence of HBV of 12.4% (64/518) and not 9.1% (47/518) as reported in our recent publication. In summary, use of HBV DNA increased the prevalence of HBV in 3.3%.

The clinical impact of OBI among HIV-infected patients is still unclear, but hepatic flares and even fatal reactivation of HBV infection following severe immunodeficiency and/or lamivudine withdrawal have been described in HIV co-infected patients [7, 32, 33]. Concern for potential negative impact of persistent OBI among HIV infected patients in Mozambique has received new attention given the high prevalence of HBV and HIV [16]. Indeed the prevalence of HIV in Mozambique increased from 11.5% in 2009 to 13.2% in 2015 and represents one of the worst epidemics in the world [16].

The frequency of anti-HBc alone found in this study was 45.2% (213/471), similar to that reported among blood donors in Mozambique [20]**.** However only 8.3% of these patients had OBI, showing a weak value of this serological marker to predict OBI. However, if we consider that more than 1.5 million of people are infected by HIV in Mozambique, it is unlikely that molecular testing could be offered routinely for such a large number of HIV infected patients and the use of anti-HBc alone in the absence of molecular testing may overestimate the prevalence of HBV (given that 45% had anti-HBc but only 8% had detectable DNA).

CD4^+^ T cells count was similar in all study groups, which corroborates finding from previous studies that showed no association between CD4^+^ T cells count and OBI in HIV patients [34].

Our data shows that most HIV infected patients with OBI had a low viremia, which was not surprising as low viremia is a hallmark of OBI [3] and similar findings were reported in other countries [5, 9, 26, 34]. In OBI, viremia is considered low due to different factors including a viral suppression resulting from strong host response occurring during acute or chronic infection [35]. Notably, 11.8% of patients with OBI had HBV DNA levels, > 1,000 copies/mL, representing a group of patients at higher risk of deterioration of liver disease as previously shown [36]. As in other studies, there was no evidence of high alanine aminotransferase (ALT) levels in patients with OBI [26, 37]. Of note, frequency of patients with significant fibrosis according to measurement of APRI and FIB-4 scores was higher in patients with OBI, however, due to the transversal design of our study we are unable to conclude that HIV infected patients with OBI are at high risk of progressing to liver disease. Current data on the impact of OBI in liver disease in HIV infected are inconclusive [1] with some showing higher risk of hepatic flares [7] and other showing no impact [25, 34]. This highlight that longitudinal studies are urgently needed to address this issue. However, administration of ART regimens containing TDF plus 3TC or FTC in immunosuppressed patients with OBI is considered the most effective intervention for long term prevention of liver inflammation [9]. Mozambique has endorsed the WHO recommendation for immediate initiation of ART in patients co-infected with HIV and HBV, and discussion for the routine testing of HBsAg in all HIV infected patients are ongoing. However, we anticipate that a significant number of HIV patients who have OBI will not be identified using the routinely available serological assays for detection of HBV in limited resource settings, which is the HBsAg marker. Consequently, these patients may be falsely classified as negative using HBsAg detection alone and subsequently at risk for unresolved HBV infection and liver inflammation [5, 9, 26, 27]. Moreover, it is less likely that molecular screening of HBV will be routinely available in many developing countries in the near future, including in Mozambique.

Most of participants in this study were female, which is not surprising as is well known that women attend more frequently health facilities as compared to men.

We would like to acknowledge additional limitations of our study. First, we acknowledge a lack of severely immunosuppressed HIV patients in our sample, which did not allow us to understand the profile of OBI in these patients and the transversal design of our study limited our ability to determine the impact of OBI in the progression to liver disease. Second, we acknowledge that the limit of detection of our assay did not allow us to detect presence of virus with viral load below 20 UI/ml. Lastly, sequencing and genotyping of HBV from patients with OBI was not performed due to a lack of resources, for this reason we are not able to ascertain if there are genetic difference in the genome of the virus from infected patients with and without OBI.

## Conclusion

Our study demonstrated that OBI is prevalent among HIV infected patients in Mozambique, suggesting that a significant number of HIV infected patients who are co-infected with HBV will not be identified using commonly used serological assays. This potentially could be a reason for missing an opportunity for immediate initiation of ART. The presence of HBV DNA level in 11.8% and higher frequencies of significant fibrosis and cirrhosis in patients with OBI, suggests that these patients are at higher risk of progression to liver disease. In conclusion, our data shows that urgent interventions are needed to address this problem in settings such as Mozambique.

## Supporting information

S1 File Questionnaire. (DOCX)

S2 File Clinical signs of liver disease in HIV patients with HBsAg negative. (XLS)
