# Supplementary material for: First report of occult hepatitis B infection among ART naïve HIV seropositive individuals in Maputo, Mozambique
Source: PLoS One. 2018 Jan 10;13(1):e0190775. doi: 10.1371/journal.pone.0190775 (PMC5761887; doi:10.1371/journal.pone.0190775)
Supplement: S1 File — (DOCX) [file pone.0190775.s001.docx]

**Frequência da hepatite B oculta em doentes infectados por vírus da imunodeficiência humana (VIH) 1/2, na área de Mavalane, Cidade de Maputo, Moçambique**

**Formulário Sócio-Demográfico**

N° do Participante: HBOG-|__|__|__| Data: |__|__|-|__|__|-20|__|__|

| Iniciais do participante: ____ ____ ____ | Unidade Sanitária:___________________ |
| --- | --- |
| Nº do Processo Clínico **\|___\|___\|___\|___\|___\|___\|___\|___\|___\|___\|**  Data de nascimento (dd/mm/aa):\|___\|\|___\| /\|___\|\|___\|/1 9 \|___\|\|___\| Idade: \|___\|\|___\| anos  Sexo: Masculino ❒ Feminino ❒  Estado civil:  Solteiro(a) ❒ Casado(a) ❒ Viúvo(a) ❒ Vive maritalmente ❒ Divorciado(a) ❒    Profissão: ___________________________ Trabalhador de Saúde: Sim❒ Não ❒  Nível de escolaridade: Analfabeto❒ Primário ❒ Secundário ❒ Universitário ❒  Bairro:______________________________________N° Casa______________________  Morada (AV/Rua)_________________________________ Quarteirão________________  Ponto de referência _________________________________________________________  Telefone do participante: (+258 ) \|__\|__\|-\|__\|__\|__\|__\|__\|__\|__\|  (+258 ) \|__\|__\|-\|__\|__\|__\|__\|__\|__\|__\|  Nome da pessoa a contactar:_________________________________________________  Telefone:(+258 )\|__\|__\|-\|__\|__\|__\|__\|__\|__\|__\|, (+258 )\|__\|__\|-\|__\|__\|__\|__\|__\|__\|__\| | |

Nome do investigador _______________________ Data:|__|__|-|__|__|-|__|__|__|__|

2.Nome do digitador____________

(iniciais)

Data: |__|__|-|__|__|-|__|__|__|__|

1.Nome do digitador____________

(iniciais)

Data: |__|__|-|__|__|-|__|__|__|__|

**Formulário de Avaliação de Factores de Risco e Co-morbilidades**

**N° do Participante: HBOG - |__|__|__| Data: |__|__|-|__|__|-20|__|__|**

| **Factores de risco actuais** | **Sim** | **Não** | **Ano de início** |
| --- | --- | --- | --- |
| Consumo álcool | ❒ | ❒ | **\|__\|__\|__\|__\|** |
| Tabaco | ❒ | ❒ | **\|__\|__\|__\|__\|** |
| Consumo de drogas endovenosas | ❒ | ❒ | **\|__\|__\|__\|__\|** |
| Partilha de agulha/seringa | ❒ | ❒ | **\|__\|__\|__\|__\|** |
| Escarificações | ❒ | ❒ | **\|__\|__\|__\|__\|** |
| Tatuagens/piercing | ❒ | ❒ | **\|__\|__\|__\|__\|** |
| História de transfusão de sangue prévia | ❒ | ❒ | **\|__\|__\|__\|__\|** |
| História prévia de vacinação de hepatite B | ❒ | ❒ | **\|__\|__\|__\|__\|** |
| História familiar de hepatite B | ❒ | ❒ | **\|__\|__\|__\|__\|** |
| Parceiros ocasionais? | ❒ | ❒ | **\|__\|__\|__\|__\|** |
| Uso de preservativo | ❒ | ❒ | **\|__\|__\|__\|__\|** |
| Troca de sexo por dinheiro, bens ou serviços | ❒ | ❒ | **\|__\|__\|__\|__\|** |
| Relação sexual desprotegida nos últimos 6 meses com parceiro ocasional | ❒ | ❒ |  |
| Relação sexual desprotegida entre últimos 6 – 12 meses com parceiro ocasional | ❒ | ❒ |  |
| No de parceiros sexuais actuais | Nenhum ❒ | 1 ❒ | >=1 ❒ |
| Resultado de teste de VIH do parceiro | Positivo ❒ | Negativo❒ | Desconhecido/Não testado❒ |

| **Estado serológico e tratamentos feitos** | | |
| --- | --- | --- |
| Alguma vez fez o teste de hepatite B | □ Sim | □ Não |
| *Se sim, qual foi o resultado* | □ Positivo | □ Negativo |
| *Se positivo, fez algum tratamento* | □ Sim | □ Não |
| *Se sim, qual e desde quando?* | _____________________ | \|__\|__\|-\|__\|__\|-\|__\|__\|__\|__\| |
| Quando fez o teste de VIH | \|__\|__\|-\|__\|__\|-\|__\|__\|__\|__\| |  |
| Nessa altura apresentava alguma doença? | _________________________ |  |

| **Co-morbidades** | **Sim** | **Não** | **Ano de início** |
| --- | --- | --- | --- |
| Diabetes mellitus | ❒ | ❒ | **\|__\|__\|__\|__\|** |
| Insuficiência renal crónica em diálise renal  Doença crónica conhecida  Medicação habitual | ❒  ❒ | ❒  ❒ | **\|__\|__\|__\|__\|**  **\|__\|__\|__\|__\|** |
| Outra:___________________ | ❒ | ❒ | **\|__\|__\|__\|__\|** |

| **Exame físico sumário** | | |
| --- | --- | --- |
| Peso ___ ___ ___ . __ kg | Altura ___ ___ ___cm |  |
| TA __ __ __/__ __ __ mmHg | FC ___ ____ ___ BPM | To Axilar __ __ .__ºC |
| Icterícia | ❒ Sim | ❒ Não |
| Ascite | ❒ Sim | ❒ Não |
| Hepatomegália | ❒ Sim | ❒ Não |
| Esplenomegália | ❒ Sim | ❒ Não |

**Frequency of occult hepatitis B in HIV (1/2) infected patients in Mavalane area, Maputo City, Mozambique**

**Socio-Demographic Form**

| Participant number: HBOG-\|__\|__\|__\| | Date: \|__\|__\|-\|__\|__\|-20\|__\|__\| |
| --- | --- |
| Participant initials : ____ ____ ____ | Health Unit:______________________ |
| Patient File number **\|___\|___\|___\|___\|___\|___\|___\|___\|___\|___\|** | |
| Date of birth (dd/mm/yy):\|___\|\|___\| / \|___\|\|___\| / 1 9 \|___\|\|___\| Age: \|___\|\|___\| years  Gender: Male ❒ Female ❒  Marital status:  Single ❒ Married ❒ Widowed ❒ Common- law marriage ❒ Divorced ❒  Occupation: _________________________________ Health worker: Yes ❒ No ❒  Education level : Illiterate ❒ Primary ❒ Secundary ❒ University ❒  Neighborhood:___________________________________ House no_________________  Address (Av/Street)_______________________________ City block _______________  Reference point ___________________________________________________________  Participant telephone number : (+258 ) \|__\|__\|-\|__\|__\|__\|__\|__\|__\|__\|  (+258 ) \|__\|__\|-\|__\|__\|__\|__\|__\|__\|__\|  Name of the contact person:_________________________________________________  Telephone:(+258 )\|__\|__\|-\|__\|__\|__\|__\|__\|__\|__\|, (+258 )\|__\|__\|-\|__\|__\|__\|__\|__\|__\|__\| | |

Name of investigator ___________________________Date:|__|__|-|__|__|-|__|__|__|__|

1.Name of data collector________

(initials)

Date: |__|__|-|__|__|-|__|__|__|__|

2.Name of data collector_________

(initials)

Date: |__|__|-|__|__|-|__|__|__|__|

**Risk Factors And Comorbidities Assessment Form**

Participant number: HBOG **- |__|__|__|** Date**: |__|__|-|__|__|-20|__|__|**

| **Current Risk Factors** | **Yes** | **No** | **Start year** |
| --- | --- | --- | --- |
| Alcohol consumption | ❒ | ❒ | **\|__\|__\|__\|__\|** |
| Tobacco | ❒ | ❒ | **\|__\|__\|__\|__\|** |
| Intravenous drug use | ❒ | ❒ | **\|__\|__\|__\|__\|** |
| Needle/ syringe sharing | ❒ | ❒ | **\|__\|__\|__\|__\|** |
| Traditional tattooing | ❒ | ❒ | **\|__\|__\|__\|__\|** |
| Tattoos/piercings | ❒ | ❒ | **\|__\|__\|__\|__\|** |
| History of previous blood transfusion | ❒ | ❒ | **\|__\|__\|__\|__\|** |
| Previous history of hepatitis B vaccination | ❒ | ❒ | **\|__\|__\|__\|__\|** |
| Family history of hepatitis B | ❒ | ❒ | **\|__\|__\|__\|__\|** |
| Occasional partners | ❒ | ❒ | **\|__\|__\|__\|__\|** |
| Condom use | ❒ | ❒ | **\|__\|__\|__\|__\|** |
| Sex in exchange for money, goods or services | ❒ | ❒ | **\|__\|__\|__\|__\|** |
| Unprotected sexual intercourse in the last 6 months with an occasional partner | ❒ | ❒ |  |
| Unprotected sexual intercourse between last 6 - 12 months with occasional partner | ❒ | ❒ |  |
| Number of current sex partners numbers | None ❒ | 1 ❒ | >=1 ❒ |
| Partner HIV test result | Positive ❒ | Negative❒ | Unknown /not tested ❒ |

| **Serological status and previous treatments** | | |
| --- | --- | --- |
| Have you ever had the hepatitis B test | □ Yes | □ No |
| *If yes, what was the result* | □ Positive | □ Negative |
| *If positive, did you had any treatment* | □ Yes | □ No |
| *If yes, which one and when did you start?* | _________________________ | \|__\|__\|-\|__\|__\|-\|__\|__\|__\|__\| |
| When did you take the HIV test? | \|__\|__\|-\|__\|__\|-\|__\|__\|__\|__\| |  |
| Did you have any illness/symptoms at that time? | _________________________ |  |

| **Comorbidities** | **Yes** | **No** | **Start year** |
| --- | --- | --- | --- |
| Diabetes mellitus | ❒ | ❒ | **\|__\|__\|__\|__\|** |
| Chronic renal failure on dialysis  Known chronic disease  Usual medication | ❒  ❒ | ❒  ❒ | **\|__\|__\|__\|__\|**  **\|__\|__\|__\|__\|** |
| Other :___________________ | ❒ | ❒ | **\|__\|__\|__\|__\|** |

| **Summary physical examination** | | |
| --- | --- | --- |
| Weight ___ ___ ___ . __ kg | Height ___ ___ ___cm |  |
| BP __ __ __/__ __ __ mmHg | HR ___ ____ ___ BPM | Axillary Tº __ __ .__ºC |
| Jaundice | ❒ Yes | ❒ No |
| Ascites | ❒ Yes | ❒ No |
| Hepatomegaly | ❒ Yes | ❒ No |
| Splenomegaly | ❒ Yes | ❒ No |
